# A LONGITUDINAL EXAMINATION OF SEXUAL SATISFACTION ACROSS ADULTHOOD IN MIDUS

**DOI:** 10.1093/geroni/igad104.2165

**Published:** 2023-12-21

**Authors:** Riki Slayday, Martin Sliwinski, David Almeida

**Affiliations:** The Pennsylvania State University, State College, Pennsylvania, United States; Pennsylvania State University, State College, Pennsylvania, United States; The Pennsylvania State University, State College, Pennsylvania, United States

## Abstract

Sexuality is fundamental to the human experience and midlife is a transition period in adulthood where sexual changes often occur. Sexual satisfaction (SS) in midlife and older adulthood is still under studied. However, researchers have found a more pronounced decline in SS among women compared to men beginning in late midlife or early old age. The purpose of the present study was to examine how sexual satisfaction changes across adulthood (20-92 years) and at what ages women and men differ on their ratings of sexual satisfaction. Participants were from waves 1, 2, and 3 of MIDUS (n=2,348; Mage=54.34 years). SS was operationalized using “How would you rate the sexual aspect of your life these days,” with a scale from 0 to 10 where 0 means “the worst possible situation” and 10 means “the best possible situation”. Time-varying effect modeling (TVEM) revealed that there was an overall linear decline until the early 60s, then the decline steepened. Men and women did not differ from ages 20 to 44. However, around age 45, men (β=5.95; CI:5.83,6.07) began reporting significantly greater SS than women (β=5.62; CI:5.45,5.74), which lasted almost four decades. At age 84, women (β=3.36; CI:2.94,3.78) reported significantly greater satisfaction then men (β=3.32; CI:2.89,3.74). On average, SS declined with age. Women reported lower SS than men for almost four decades (ages 40-80); however, women reported higher satisfaction than men starting in their early eighties. No studies have examined SS across adulthood or gender differences in SS using TVEM.

